# Suicide Risk and Mental Disorders

**DOI:** 10.3390/ijerph15092028

**Published:** 2018-09-17

**Authors:** Louise Brådvik

**Affiliations:** Faculty of Medicine, Department of Clinical Sciences Lund, Lund University, Lund SE 221 00, Sweden; louise@bradvik.se

Suicide is a major health problem, and the global suicide mortality rate amounts to 1.4% of all deaths worldwide. Most suicides are related to psychiatric disease, with depression, substance use disorders and psychosis being the most relevant risk factors [1]. However, anxiety, personality-, eating-, and trauma-related disorders, as well as organic mental disorders, also contribute.

Psychological autopsies from the middle of the previous century and onwards have revealed that most people who have died by suicide have suffered from mental disorders [2]. A recent figure suggests this number could be at least 90% [3,4]. On the other hand, most people with mental disorders do not die by their own hand. The risk of suicide has been estimated to be 5–8% for several mental disorders, such as depression, alcoholism and schizophrenia [5,6]. 

A stress-diathesis model has been proposed in which the risk for suicidal acts is determined not merely by a psychiatric illness (the stressor) but also by a diathesis, such as a tendency to experience more suicidal ideation and to be more likely to act on suicidal feeling [7]. Thus, suicide risk is multi-factorial. 

Research on the risk factors among people with mental disorders is urgent in the efforts to predict and prevent suicide death. In the present special issue, including 13 articles, suicide risk and mental disorders are explored from several different perspectives.

According to a review, most studies on ethnicity/immigrant status and suicide attempts showed higher rates among immigrants as compared to the native population [8], though the reverse was found in a few cases. Risk factors were found to be: language barriers, worrying about family back home and separation from family, often leading to hopelessness, depression, and anxiety. Furthermore, the lack of information on the health care system, loss of status, loss of social network, as well as acculturation (when an individual acquires attitudes, etc. from a different country) were identified as potential triggers. 

Adverse childhood experiences (ACE) have been implicated in a range of negative health outcomes in adulthood, including mental disorders and suicide death. One paper explores the relationship between ACE and hospital-treated self-harm in Glasgow [9]. First-time and repeat self-harm were compared, including mental health, psychosocial measures, and attachment style. However, only ACE along with female gender and depressive symptoms differentiated between first-time and repeat self-harm. Participants with 4+ ACE were significantly more likely to repeat. There were no differentiating factors in the male group. The findings stress the importance of ACE as a risk factor for suicidal behaviour, but more research is needed for the male group.

Suicide prevention efforts often depend on the disclosure of suicidal ideation (SI), an early step in the suicidal process. Data from the Dutch cross-sectional survey Health Monitor 2016 was used in one study [10]. Of the adult participants, 5% reported SI and nearly half of those had not disclosed their SI. Thus, non-disclosure was a substantial problem, which may interfere with the efforts to prevent suicide. Adults who did not disclose SI were more likely to have few social contacts, while they were less likely to report poor mental health and frequent SI. There was no significant association found between emotional loneliness or marital status and SI. The availability of social support rather than the intimacy of relationship was suggested. In line with these findings, some subjects, in a long-term follow-up of former suicide attempters [11], pointed out that the decision to continue living was a very private one, not necessarily communicated with others. The problem of disclosure should be considered in suicide prevention strategies.

Depression is strongly related to both suicidal ideation and attempt, but it lacks specificity as a predictor, and little is known about the characteristics that increase the risk of suicide among people with depression. As part of the Australian Rural Mental Health Study, the relationship between depression and suicidal behaviour was investigated [12]. Out of 1051 participants, 364 reported life-time depression. Of these, 48% reported life-time suicidal ideation and 16% reported a life-time suicide attempt. The severity of depression was a significant correlate of suicidality in both men and women, but suicide attempts were significantly more common among females with a younger age of depression onset, and a higher number of psychiatric comorbidities. No additional factors were found for males, which agrees with the study on ACE above [9]. Prediction may be more difficult in men, and they more often die by their own hand.

Depression and substance use disorders, mostly alcohol, are the most prevalent diagnoses among suicide victims [4]. Furthermore, comorbid disorders, are associated with a high suicide risk [1,13,14]. One paper in the present issue explores comorbid disorders and suicide risk in severe depression/melancholia (major depressive disorder with melancholic and/or psychotic features-MDD-M/P) [15]. Hospitalized patients with MDD-M/P who later died by suicide were compared with matched controls who died a natural death (or in a few cases survived) in a long-term follow-up of almost 60 years. Comorbidity was found to be common for obsessive compulsive symptomatology, anxiety and schizophrenia but not alcohol (partly due to fact that all patients were chosen to have depression as first diagnoses). However, counter-intuitively, there was no difference in comorbidity of any kind between suicide victims and controls. Thus, in contrast to other studies on depression and suicide risk, comorbidity does not appear to increase the risk in MDD-M/P. This raises the possibility that MDD-M/P per se has a high risk of suicide, which is not increased by comorbid disorders. The evaluation of MDD-M/P appears important in evaluating suicide risk.

Implementation of suicide prevention guidelines in mental healthcare institutions (MHI) is important in the efforts to reduce suicide rates [16]. In the Netherlands, a prospective cohort study was performed to change ten domains’ policies and practices. There was a significant improvement in four domains; the development of an organizational suicide prevention policy; monitoring and trend-analysis of suicides numbers; evaluations after suicide; and clinician training. However, no improvement was measured on the other domains, such as a routine assessment of suicidality in all patients, safety planning, and involving next of kin and carers. Furthermore, there was a marked practice variation between MHIs. The implementation of guidelines is important, and means to improve other relevant domains should be developed, including reduced differences between MHIs.

An assessment of the use of media reporting recommendations by the World Health Organization in suicide news published in the most influential media sources was performed in China during a twelve-year time span [17]. Three recommendations were applied in most suicide stories, but four recommendations were rarely applied, one of them being information about where to seek help and linking the suicide event to mental disorders.

In some countries, assisted suicide (AS) is allowed in severe medical illness. In Switzerland the practice of lay right to-die (RTSD) organizing AS is tolerated by the state, and lay involvement is unique for the country. A paper from Basel University Hospital [18], deals with handling the twofold and divergent duties: assisted suicide and caring for patients with suicidal thoughts, or after a suicide attempt. There has been a strong commitment to suicide prevention in Switzerland despite the tolerance of AS. The legal framework and ethical guidelines are discussed, and practical examples are presented, where the patient’s mental health and capability of decision are considered. The evaluation of mental illness and importance of treatment are also stressed, with the possibility to change the patient’s attitude to suicide. 

The present special issue on ‘suicide risk and mental disorders’ reflects several aspects of prediction, including ethnicity/immigrant status, adverse childhood experiences, severity of depression and comorbidity of mental disorders. Other papers deal with internet addiction [19], risk of suicide in type 2 diabetes [20], and risk factors for suicidal ideation in rural and urban areas [21]. Suicide risk is multi-factorial and the stress (mental illness)—diatheses (tendency to experience suicidal ideation and act on suicidal feelings) model is applicable, with several examples of the latter described in the present special issue. Furthermore, other important aspects of suicide risk should be considered, such as disclosure of suicidal ideation, suicide prevention guidelines in mental healthcare institutions, and whether the recommendation of the media reporting of suicide events are followed. One article describes involvement of family members after sentinel events [22], such as suicide death. Finally, ethical issues are discussed. In an era when assisted suicide is tolerated for medical illness, it is vital to remain committed to suicide prevention in mental disorders. The decisional capacity of people with mental illness is distorted, and this illness, as well as suicidal ideation, are often reversible. 

Depression is a common thread throughout the articles, also known to be the most common disorder among people who die by suicide. In a review a few years ago, the risk factors in this disorder were family history of psychiatric disorders, male gender, suicide attempts, more severe depression, hopelessness and comorbidity [23]. Interestingly, the review on suicide risk in depression could only include 19 studies (28 papers), which is a surprisingly low number considering the high suicide risk in the disorder. 

More research is needed to improve the prediction of suicide in mental disorders, along with more effective implementation of preventative measures. The present special issue shows some important examples of risk factors and constructive ways to go forward in the efforts to prevent suicide deaths.
